# ICF Linking and Cognitive Interviewing Are Complementary Methods for Optimizing Content Validity of Outcome Measures: An Integrated Methods Review

**DOI:** 10.3389/fresc.2021.702596

**Published:** 2021-10-14

**Authors:** Joy C. MacDermid

**Affiliations:** ^1^Department of Surgery, School of Physical Therapy, Western University, London, ON, Canada; ^2^Hand and Upper Limb Centre, St. Joseph's Health Centre, London, ON, Canada

**Keywords:** ICF, linking, content validity, PROM, cognitive interviewing, methods, outcome measures

## Abstract

Content validity is a fundamental requirement of outcome measures. After reviewing operational needs and existing definitions, content validity we as defined as: the extent to which a measure provides a comprehensive and true assessment of the key relevant elements of a specified construct or attribute across a defined range, clearly and equitably for a stated target audience and context. ICF linkage rules from 2002, 2005, and 2019 have provide increasingly clear processes for describing and evaluating content of outcome measures. ICF Core Sets provide international reference standards of the core constructs of importance for different health conditions. Both are important as reference standards during content validation. To summarize their use as reference standards, the following summary indicators were proposed: (1) Measure to ICF linkage, (2) Measure to (Brief or Comprehensive) Core Set Absolute Linkage, (3) Measure to (Brief or Comprehensive) Core Set Unique Linkage, (4) Core Set Representation, and (5) Core Set Unique Disability Representation. Methods to assess how respondents engage with content are needed to complement ICF-linking. Cognitive interviewing is an ideal method since it used to explore how respondents interpret and calibrate response to individual items on an outcome measure. We proposed a framework for classifying these responses: Clarity/Comprehension, Relevance, Inadequate response definition, Reference Point, Perspective modification, and Calibration Across Items. Our analysis of 24 manuscripts that used ICF linking for content validation since updated linking rules were published found that authors typically used linking to validate existing measures, involved multiple raters, used 2005 linking rules, summarized content at a concept level (e.g., impairment, activity, participation) and/or use core sets as a reference standard. Infrequently, ICF linking was used to create item pools/conceptual frameworks for new measures, applied the full scope of the 2019 linking rules, used summary indicators, or integrated ICF-linking with qualitative methods like cognitive interviews. We conclude that ICF linkage is a powerful tool for content validity during development or validation of PROM. Best practices include use of updated ICF linking rules, triangulation of ICF linking with participant assessments of clarity and relevance preferably obtained using cognitive interview methods, and application of defined summary indicators.

## Introduction

The issue of content validity of health outcome measures is the most critical, and most neglected area of clinical measurement science. Content validity is important for all health outcome measures and is especially complex to measure for patient-reported outcome measures (PROM) since how potential respondents interact with items depends on a variety of factors related to the respondent e.g., age, language, culture, lifestyle, life experience, health; and factors related to the PROM e.g., content, clarity, and comprehensiveness. Development of methods for content validation support more rigourous development of new PROM and evaluation of existing PROM. Progress in different aspects of content validity and ICF linking has been evident in recent years. For this reason, an integrated narrative review that focuses on methods for using ICF linking in content validation is one way to bring together emerging work with a view to greater clarity and rigor in content validity research.

The purposes of this paper are:

1. To describe previous content validity definitions and propose a more comprehensive operational definition
2. To discuss how ICF linking can be used to support content validation
  a. To provide simple indicators that can be used to summarize how PROM items codes relate to ICF and relevant ICF Core Sets
3. To describe how cognitive interviewing complements ICF linking
  a. To provide summary indicators for describing sources of potential errors or cognitive dissonance as respondents interpret and respond to PROM instructions or items
4. To describe, through a structured review process, how ICF linking has been used in the development or evaluation of item pools for PROM since the updated 2016 ICF linking rules were published.

## Content Validity Definitions

Content validity has been defined by multiple authors with varying elements. We located published definitions of content validity and listed below some of the key existing definitions. This is not an exhaustive list of all known published definitions but illustrates that there is some shared vision of what constitutes content validity in prior literature, but also that definitions differ in their focus on relevance, range, clarity, and representation of the construct as key elements of content validity. Based on core constructs from different definitions and methodologies used to assess content validity we have constructed an operational definition (Table 1).

**Table 1:** Definition of content validity.

The definition of content validity “*the extent to which a measure provides a comprehensive and true assessment of the key relevant elements of a specified construct or attribute across a defined range, clearly and equitably for a stated target audience and context*”

1. The degree to which elements of an assessment instrument are relevant to, and representative of the targeted construct, for a particular assessment purpose (1)
2. The degree to which an instrument has an appropriate sample of items for the construct being measured (2)
3. Whether or not the items sampled for inclusion on the tool adequately represent the domain of content addressed by the instrument (3)
4. The extent to which an instrument adequately samples the research domain of interest when attempting to measure phenomena (4)
5. The extent to which a scale or questionnaire represents the most relevant and important aspects of a concept in the context of a given measurement application (PROMIS consensus) (5)
6. The degree to which a sample of items, taken together, constitute an adequate operational definition of a construct (6)
7. The degree to which the content of an instrument is an adequate reflection of the construct to be measured (7)
8. The extent to which a subject's responses to the items of a test may be considered to be a representative sample of his responses to a real or hypothetical universe of situations which together constitute the area of concern to the person interpreting the test (8).
9. Whether or not the items sampled for inclusion on the tool adequately represent the domain of content addressed by the instrument (3).

**Figure 1:**
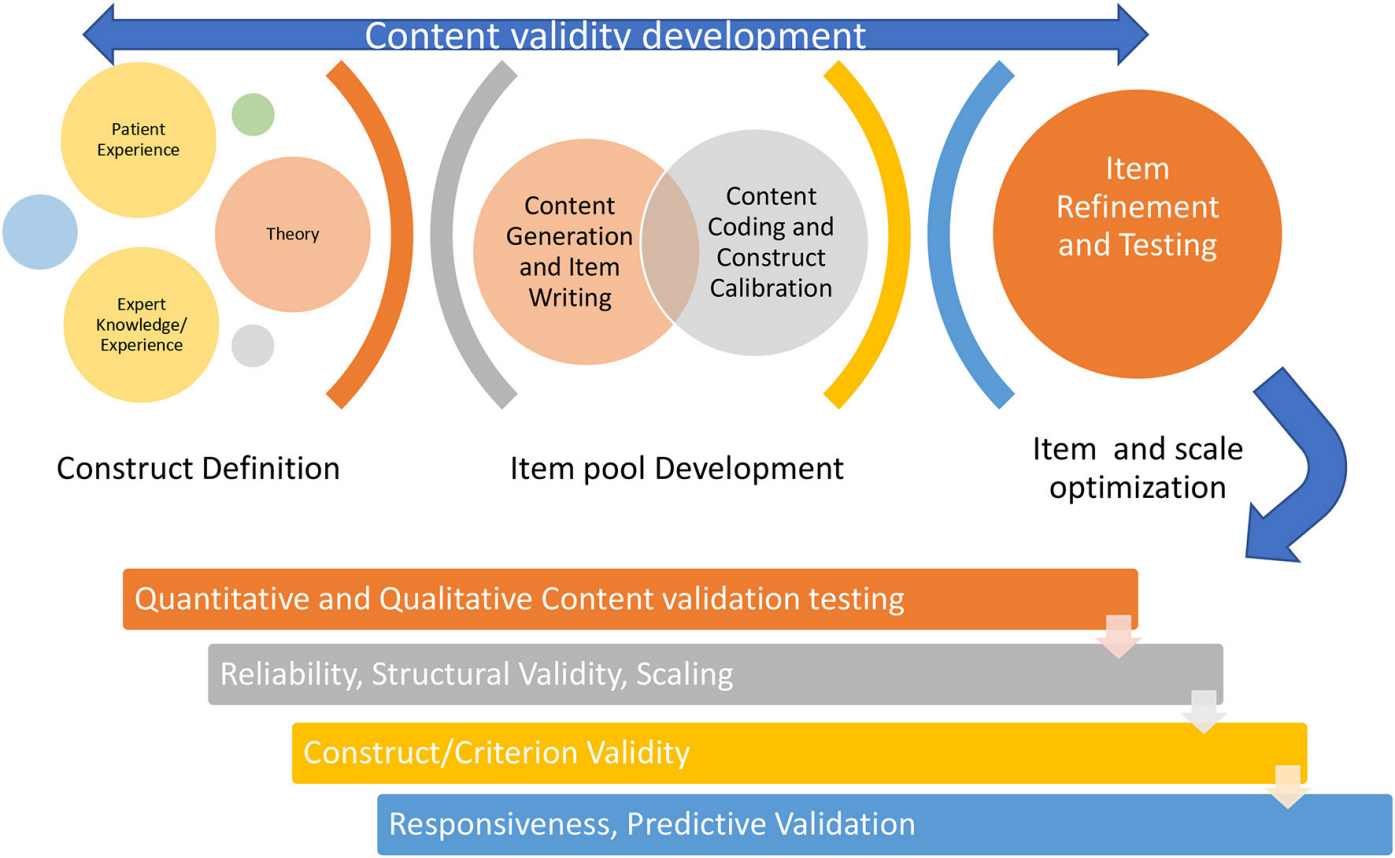
Content validation is a key foundation when developing and evaluating PROM.

Ideally content validity is well-attended to during development of a PROM since content validity requires careful conceptualization of the construct and potential domains during item generation and preliminary testing (Figure 1). Basic science, theory, and quantitative or qualitative empirical studies on the experiences of people living with impairment or disability can inform item generation (9). Developers often use or adapt items from pre-existing PROM. Clinical experts can provide expert knowledge of the health condition mechanisms and impacts and are ideally suited to judge whether a PROM addresses the nature and range of health manifestations of a given health condition, or the attribute being assessed. Social media scraping and observational studies of behavior can also inform the item generation but are less commonly used than methods involving patient/expert interviews or surveys. The International Society for Pharmacoeconomics and Outcomes Research (ISPOR) Board of Directors produced a report on “Patient Reported Outcomes (PRO) Content Validity Good Research Practices” that emphasized the importance of qualitative approaches to item generation (10). It stated that important steps include: having a framework, coding system and training of coders to optimize the rigor of moving from qualitative interviews to PROM items. The PROMIS group (5) emphasized that understanding content validity includes: (1) specifying the concept the scale is intended to represent; (2) scaling the concept's various components and items; and (3) defining the PRO measure's intended purpose, the opinions of patients about whether the item is relevant to them and the clarity of how the item is framed.

It is a responsibility of the instrument developer to develop/test items that are representative of the target construct/attribute and that are relevant for a wide array of users. Items should cover a sufficient range, be equitable and generalizable. It is important to consider differences due to gender, culture, age, differing levels of health literacy, and factors that might affect how a construct manifests or how an item measures the intended construct. Yet these issues are often underexplored and vaguely reported during content validation. Achieving the best pool of item is a challenging and iterative process. While Figure 1 suggests a sequence, the ordering of steps, optimal number of iterations varies, and processes can vary at each state. Consultation with the target population, clinical experts and method experts can be used to refine definitions of the construct, target audience and the item pool. In our experience it is important to include both quantitative and qualitative methods during development of items. The patient perspective is the most important one since PROMs represent the patient view and give patients a voice in outcome evaluations. Experts in clinical measurement methods and clinical experts can contribute insights that are unique and complementary to that provided by patients.

The investigation of content validity often occurs, or is extended, after a PROM is made available for public use. This is important since content validity may have been insufficiently reported by the original developers, can vary by target audience or be different across different contexts/cultures. A clear operational definition of content validity and tools to assess content validity can facilitate rigorous evaluation of existing PROMs and can inform what methods are needed to assess aspects of content validity. For example, COSMIN suggested 10 criteria for evaluation of content validity: 5 are allocated to relevance, one to comprehensiveness and 4 to comprehensibility (11).

Content validity of PROM is dependent on the item pool being rich, diverse, and yet specific to the intended construct. Item generation processes can use patient interviews, other PROMs, and expert opinion as sources. In some cases, developers start with a pre-defined construct and define items that fit within that construct. In other cases, the patient and expert opinions are used to define a model or definition of the construct being measured, before proceeding to item generation. Content must be interpreted and classified in a way that leads to specific items and a structure (unidimensional or multiple subscales). Content validity indices use survey methods and percentage indicators to summarize how respondents rate the relevance of items (4, 6, 12, 13). Clinimetric methods consider importance and frequency ratings as indicators of relevancy (14, 15). Some authors use qualitative approaches, to explore relevance and ease of comprehension during development (10, 16–18). It is likely that integration of multiple methods is needed to determine whether items represent the spectrum, context, and features of the intended construct, since different methods have strengths and limitations. Unfortunately, many developers fail to report how qualitative interviews or opinions directly led to generation of items. Common areas that are under-reported are how the construct was defined, how the subcomponents of that construct were defined and how the items were generated to reflect the appropriate dimensions and weighting of elements of that construct. Methodologists have made some steps toward clarifying methods, such as those reported by the COSMIN group (7, 11). ICF linking rules which provide a framework and method for describing and classifying content about functioning, disability and health are an important tool for PROM developers and evaluators (19–25).

Psychometric studies of PROM often focus on quantitative measurement properties like reliability, structural or construct validity and responsiveness. As a result, systematic reviews of PROM often fail to address content validity or find it lacking for existing PROM. However, investigating psychometric properties of PROM that have not been adequately subject to content validity is problematic. Items with poor clarity contribute to random error which limits reliability and makes it more difficult to detect true changes (responsiveness) or real relationships between variables (construct validity). Proof of reliability does not mean that the intended construct intended is being measured, it only means that scores are stable. Analyses like factor analysis or Rasch analysis are likely to demonstrate poor model fit if content validity is inadequate. Therefore, content validity should be considered an essential prerequisite for investigation of psychometric properties.

Failure to establish content validity can have negative consequences on health research since inadequate content validity undermines the validity of the conclusions. We may fail to detect treatment effects if PROM do not capture the elements that the treatment is targeting. Conversely, if a PROM assesses different constructs than intended, this can lead to false conclusions about the mechanisms of action of an intervention. Limited content validity can impede the progress of health research by confounding our understanding of phenomena, allowing inaccurate attribution of causation, failing to identify effective interventions, or accepting interventions based on flawed assumptions.

For all the reasons above greater attention to the methods of content validity is important for health research. The focus of this integrative review is to focus on the use of ICF linking to describe the content of PROMs and how it is complemented by exploring how patients interact with content using cognitive interviewing.

## ICF Linking and Summary Indicators

Icf provides a conceptual framework that considers body structure/function (impairments), activity (limitations) and participation (restrictions) (26). These interacting domains of health can be modified by personal and environmental factors (27). ICF also provides a hierarchical coding system where body structure (s), body function (b), disability (d), and environmental (e) factors codes can be used to describe the aspects of disability, functioning and health that are affected using a common language. Like any language, ICF can support an unlimited number of applications. Clinical measurement research is just one of the many uses of ICF linking. ICF linking was defined in 2002 (28) and has been refined in 2005 (21) and 2019 (22) in update publications (published online in 2016). See Table 2 to review the progression of these linking rules.

**Table 2:** Evolution of ICF linking rules developed by Cieza et al. (23).

**2002 linking rules (23)**
1. Before one links health-status measures to the ICF categories, one should have acquired good knowledge of the conceptual and taxonomical fundament s of the ICF, as well as of the chapters, domains, and categories of the detailed classification, including definitions 2. Each item of a health-status measure should be linked to the most precise ICF category 3. If a single item encompasses different constructs, the information in each construct should be linked 4. All constructs of the item to be linked have to be highlighted (e.g., bold) 5. The response options of an item are linked if they refer to additional constructs 6. If the content of an item is not explicitly named in the corresponding ICF category, then the “other specified” option at the third and fourth coding level of the ICF classification is linked. The additional information not covered by the ICF classification is documented. Two special cases are to be distinguished within this rule: a) When the ‘other specified' option in the two-level classification is not available, then the ‘other specified and unspecified' option is linked. The additional information not covered by the ICF will be documented b) When the content of an item is not explicitly named in the corresponding ICF category, but at the same time is included in the ICF-category, then the item is linked to this ICF category, and the additional information not explicitly named by the ICF is documented 7. If the content of an item is more general than the corresponding ICF category, then the code of the higher level is linked 8. If the content of an item is more general than any ICF category but otherwise the item specifies by examples partial aspects of the concept contained in one or more ICF categories, then the “unspecified” option of the ICF classification is linked (Code 99 for the second coding level, Code 9 for third and fourth coding levels). As statement or part of an item will be considered an example when it is introduced with “e.g.,” appears between parenthesizes, is introduced with “for example,” or with “such as” 9. If the information provided by the item is not sufficient for making a decision about which ICF category the item should be linked to, this item is assigned nd (not definable) 10. If an item is not contained in the ICF classification, then this item is assigned nc (not covered by ICF)
Linking rules updated in 2005 (21)
1. Before one links meaningful concepts to the ICF categories, one should have acquired good knowledge of the conceptual and taxonomical fundaments of the ICF, as well as of the chapters, domains, and categories of the detailed classification, including definitions 2. Each meaningful concept is linked to the most precise ICF category 3. Do not use the so-called “other specified” ICF categories, which are uniquely identified by the final code 8. If the content of a meaningful concept is not explicitly named in the corresponding ICF category, the additional information not explicitly named in the ICF is documented 4. Do not use the so-called “unspecified” ICF categories, which are uniquely identified by the final code 9 but the lower-level category 5. If the information provided by the meaningful concept is not sufficient for making a decision about the most precise ICF category it should be linked to, the meaningful concept is assigned nd (not definable) Special cases of this rule: a. Meaningful concepts referring to health, physical health or mental (emotional) health in general, are assigned nd-gh, nd-ph, or nd-mh (not definable-general health, not definable-physical health, not definable-mental health), respectively. Meaningful concepts referring to quality of life in general are assigned nd-qol (not definable-quality of life) 6. If the meaningful concept is not contained in the ICF, but it is clearly a personal factor as defined in the ICF, the meaningful concept will be assigned pf (personal factor). Personal factors are defined in the ICF as follows: “The particular background of an individual's life and living and comprise features of the individual that are not part of a health condition or health states. These factors may include gender, race, age, other health conditions, fitness, lifestyle, habits, upbringing, coping styles, social background, education, profession, past and current experience (past life events and on current events), overall behavior pattern and character style, individual psychological assets and other characteristics, all or any of which may play a role in disability at any level” 7. If the meaningful concept is not contained in the ICF and it is clearly not a personal factor, this meaningful concept is assigned nc (not covered by ICF) 8. If the meaningful concept refers to a diagnosis or a health condition, the meaningful concept will be assigned hc (health condition) Note some specific rules for health status instrument clarified rules on linking of response items and examples within items were included in this paper in a separate list.
2019 linking rules published online in and in print (22)
1. Acquire good knowledge of the conceptual and taxonomical fundamentals of the ICF, as well as of the chapters, domains, and categories of the detailed classification, including definitions before starting to link meaningful concepts to the ICF categories 2. Identify the purpose of the information to be linked by answering the question What is this piece of information about? or What is this item about? The answer to these questions will help to identify the main concept(s) most relevant to be linked to the ICF 3. Identify any additional concepts contained in the piece of information in addition to the main concept(s) already identified in the previous step 4. Identify and document the perspective taken on within a certain piece of information when linking it to the ICF (Appraisal, Needs, or dependency) 5. Identify and document the categorization of the response options. Take into consideration the most frequently used approaches as listed in Table 3 (Intensity, Frequency, Duration, Confirmation or agreement, Qualitative attributes). Note: this rule applies only to instruments, questionnaires, assessments, or tests that contain response options 6. Link all meaningful concepts, the most relevant and additional ones, to the most precise ICF category 7. Use “other specified” or “unspecified” ICF categories as appropriate At the end of the chapter, and at the end of each embedded set of third- or fourth level ICF categories, there are categories with the final code number 8 for “other specified” and 9 for “unspecified”“8” is to be used when the concept is not contained within any of the other specific categories at the respective level of a chapter. The additional information is documented after the ICF code “9” is used when the concept to be linked fits within a given chapter but there is not sufficient information at hand to assign it to a specific ICF category 8. If the information provided by the meaningful concept is not sufficient for making a decision about the most precise ICF category, assign the concept to nd (not definable) Concepts referring to health in general, physical health or mental (emotional) health in general, are assigned nd-gh, nd-ph or nd-mh (not definable-general health, not definable-physical health, not definable-mental health), respectively, as well as to disability in general (nd-dis), functioning (nd-func), or a child's development (nd-dev)
9. If the meaningful concept is not contained in the ICF but is clearly a personal factor as defined in the ICF, assign the meaningful concept to pf (personal factors) 10. If the meaningful concept is not contained in the ICF, assign this meaningful concept to nc (not covered) Further specifications: Meaningful concepts referring to a diagnosis or health condition are assigned to nc-hc (not covered-health condition). Meaningful concepts referring to quality of life or life in general are assigned nc-qol (not covered-quality of life)

The linking rules updates build on each other, while maintaining a consistent approach. Consistent across each version are rules stating that people should have content knowledge to classify content and that content should be linked as specifically and precisely as possible within the ICF hierarchical classification system. In some items, this result in codes that are broad, even at the chapter level, if the item being evaluated is posed at a very broad level. In other cases, a very specific code may be assigned, at a 3rd or 4th level, if an item has a narrower focus. Greater specificity is indicated by codes with more numbers that reflect deeper level codes and more specific code definitions. In the 2005 update the rules around how to deal with content that is difficult to code including unspecified, undefined, and global terms like quality-of-life were clarified. In 2019 there were additions to the rules to focus on the perspective taken (Appraisal, Needs, or dependency), which is an important aspect of a PROM since it focuses on the cognitive evaluation requested within an item. A substantial clarification in the 2019 update was how to deal with response options (coded as Intensity, Frequency, Duration, Confirmation or agreement, Qualitative attributes). This was an important addition since older ICF linking studies usually ignored response options. Response options are central to how patients are asked to calibrate their responses. Therefore, the 2019 linking rules have important additions that can provide more detailed description of PROM content validity.

The volume of ICF codes can be unwieldy, which may act as a barrier to usage in many applications of ICF. To address this barrier, ICF Core Sets have been developed through a rigourous process of international consensus informed by research evidence and patient/expert experiences and priorities (29, 30). The process by which the Core Sets were developed includes literature review, patient surveys and expert surveys, culminating in an international consensus conference that achieves agreement on the most salient content for health conditions based on the discussion and voting of a multidisciplinary international group who can envision many different applications and who represent many unique perspectives. Core sets have now been established for many conditions in 7 different domains covering different health conditions and contexts (https://www.icf-research-branch.org/icf-core-sets). Given that a comparison gold standard is elusive when evaluating PROMS, ICF Core Sets provide an important reference standard for the most salient content for PROM addressing functioning, disability, and health. ICF linking can be particularly useful for disease-specific PROM where there are relevant Core Sets since they act as an international reference standard for that health condition. Core sets and ICF linking are less suited for categorizing abstract concepts like emotions/attitudes, life experience, PROM that explore a single construct (e.g., pain or sleep) or concepts not covered by ICF.

A challenge that we experienced in interpreting ICF linking was how to summarize the large volume of information from our raw mapping code lists that often contain many items and codes. Therefore, we proposed simple summary statistics (Table 3) that can be used to augment other descriptive analyses such as mapping items to chapters, mapping codes to a theoretical framework, or mapping items to ICF conceptual domains. The defined set of indicators are a set of simple summary indicators that quantify the extent to which items can be coded to ICF, linked to the core sets, and represent the core sets. Recognizing that a common shared goal of ICF and some PROMs is to describe disability, a summary indicator that focused on disability content was also proposed (Table 3). We have used these indicators and found them to be helpful in describing or comparing PROM (31–33).

**Table 3:** ICF linkage summary indicators.

**ICF linkage summary indicators**
Raters can describe the content of an outcome measure using the instructions/training and established linking rules (21–23) and any further updates established by the ICF branch to select the ICF codes that best represent the content of items/measures. This content coding can then be summarized by the following indicators that compare the item/test codes to the ICF or its relevant subset Core Sets These indicators summarize codable content. Only codes are counted; “not codable” codes are reported as defined by ICF linking rules but are not included in the summary indicators below. It is useful to describe the number of codes and the distribution, e.g., by chapters or domains (structure, function, activity, participation, personal factors, environmental factors, health conditions) in addition to the summary terms below. These are intended to be descriptive summary that can be used to compare items/measures and their relationship to ICF overall to core sets but should be used in combination with other descriptive strategies to fully describe or compare measures in terms of content validity v
**Measure to ICF linkage:** This is the percentage of items from a measure that can be linked to ICF codes. This represents the extent to which content of a measure can be expressed in ICF codes
$=\frac{\text{The number of items linked to at least 1 ICF code}}{\text{Total number of items on the measure}}\times 100\%$
**Measure to (brief or comprehensive) core set absolute linkage:** This is the percentage of items from a measure that could be linked to ICF codes that appear on a relevant Brief or Comprehensive Core Set
$=\frac{\text{Number of linked to a code(s) appearing in the CoreSet}}{\text{Total number of items on the measure}}\times 100\%$
**Measure to (brief or comprehensive) core set unique linkage:** This is the percentage of items from a measure that could be linked to unique ICF codes and represents the extent to which the items of a measure represent different content from the core set. Once an item is coded to a core set item, additional items that code to that same code are not counted again
$=\frac{\text{Number of item that are linked to Unique codes in Core Set}}{\text{Total number of items on the Scale}}\times 100$
**Core set representation:** This is the percentage of core set codes that are covered when the measure's items are linked to ICF codes. This represents the extent to which the entire scope of content defined by the core set is represented on the measure
$=\frac{\text{Number of unique ICF codes from the measure that appear in the CoreSet}}{\text{Total number of codes in the (Brief or Comprehensive) CoreSet}}\times 100\%$
**Core set unique disability representation:** This is the percentage of unique core set disability codes that are covered when the measure's items are linked to ICF codes. For Patient-Reported Outcome (PROs) Measures that were designed to measure disability, it can be important to determine the extent to which they measure this aspect of content. This represents the extent to which the disability codes defined by the core set are represented on the measure. Once an item is coded to a core set disability code, additional items that code to that same code are not counted again
$=\frac{\text{Number of unique (d)codes from the measure that appear in the Core Set}}{\text{Total number of disability codes in the (Brief or Comprehensive) CoreSet}}\times 100\%$

## Cognitive Interviewing Explores How Respondents Interact With Content

Cognitive interviewing is a semi-structured interview process that explores how individuals understand, mentally process and respond to survey instructions, items and response options; and whether an individual perceives the items/measure as reflecting the intended construct or attribute being assessed (17, 34–36). Whereas, ICF linking is designed to describe and classify content, cognitive interviews are designed to explore how respondents interact with the content. Thus, they are complementary methods. Cognitive interviewing uses a semi-structured qualitative interview with think aloud and probing approaches to explore how potential respondents understand, calibrate, and respond to instructions and items on a PROM. More detailed description of these methods has been described in textbooks (17) and manuscripts (10, 17, 34, 35, 37–40).

Cognitive interviewing is ideally suited to PROM content validation since it explores the four cognitive actions involved in a response to PROM items: respondents must understand the meaning and intent of the question, they must be able to retrieve accurate information about a past or present status (rationale) or gauge their current feelings (emotional), make a judgment as to how their experience or feelings fit with the question posed, and choose an appropriate answer/response option that reflects their cognitive calibration process. Cognitive interviews also explore whether an individual perceives that the overall pool of items reflect the intended construct or attribute being assessed (15).

Cognitive interviews can generate a large amount of descriptive information that complements the large volume of information obtained from ICF linking. Therefore, we developed a guide and classification system for classifying potential sources of confusion or cognitive dissonance as respondents interpret and calibrate their response to items on a PROM. Qualitative think aloud and probing approaches are used to explore how potential respondents, content experts or measurement experts interpret the meaning of instructions, items, and responses options, and then how they calibrate their responses to items (16, 17). Sources of response error or cognitive dissonance are then classified as listed below [see web (41) or Appendix 1 for full details]. In brief, this method classifies findings from the qualitative interview into summary statistics that describe the extent to which the following issues were identified.

### Clarity/Comprehension

Refers to when the terms/words used in an item or response are ambiguous or incorrectly interpreted by respondents.

Example: “*downhearted and blue*” is used for depressive symptoms but is easily misinterpreted.

### Relevance

Refers to when an item is not relevant to respondents (e.g., task not possible or important in their circumstances).

Example: “*Washing your hair*” not relevant to bald men.

### Inadequate Response Definition

Refers to when response options provided are: 1. not mutually exhaustive or have missing options, or 2. are not matched to the questions posed.

Example: Question asks how important something is, but the response options are about frequency.

### Reference Point

Refers to when respondents have difficulty calibrating their responses to an item because their reference points have changed (e.g., response shift) or the item has unclear reference boundaries (e.g., time interval or context). Includes when respondents are unable to recall information needed to calibrate their response.

Example: “*How much have you improved*?”; respondents are unclear and may not recall prior health status (recall bias) … since when?

### Perspective Modifiers

Perspective modification occurs when items are interpreted differently by respondents based on a personal factor, life experience or environmental factor.

Example: “*Can you do your recreational activities*?”- can be very different based on activities they do.

### Calibration Across Items

Refers to when the response to one item is modified by the patient's response to a previous item.

Example: “*What is your pain at its worst*?” Respondents may score it based on what they scored for other items.

## Structured Review of Current Application of ICF Linking in Prom Content Validation

In the authors experience, ICF linking is a valuable way to code and map PROM content, and ICF core sets are valuable reference standards for evaluating content validity. To understand how ICF linking methods have used recently, we used a structured review to identify papers where ICF linking was reported in content validation (using search terms for ICF linking and content validity). We searched using Google Scholar and PubMed starting in January 2016 and ending in August 2021. We chose this timeframe to coincide with the 2016 online publication of updated linking rules that became available in print in 2019. Our goal was to establish the most current content validity research practices not to provide a comprehensive review of all studies using ICF linking. Our inclusion criteria were peer-reviewed published studies that used ICF linking to develop or evaluate items from a PROM for the purpose of content validation. Exclusion criteria: studies that used ICF linking for other purposes other than examination of content validity of PROM item, 2. when ICF linking was used to code open ended responses from PROM, 3. papers that used ICF linking to validate Core Sets not outcome measures and 4. Theses, abstracts, conference presentations, or non-peer reviewed papers. From these papers we extracted information about whether the authors used ICF linking process to inform development of items for a new measure or validation of an existing measure, which version of linking rules were used, and how data were coded and summarized. We also extracted whether ICF linking was used alone, in combination with cognitive interviewing or in combination with other methods for assessing content validity.

The findings are summarized in Table 4. We found that ICF linking has been used in a wide variety of disciplines and health conditions to assist with the development of a new PROM or validate the content validity of an existing PROM. More frequently it has been used to assess the content validity of an existing PROM, that in creation of new PROMs. Most commonly multiple raters perform the linking procedures, and there was a mixture of 2005 and 2019 rules cited. In studied citing 2019 rules, many did not report all aspects of the 2019 linking rules as findings, particularly lacking were reports of perspective and response options. Most often the data was interpreted by focusing on how the codes fell into different conceptual domains (e.g., impairment, activity, participation concepts) or ICF chapters; and summarized in charts that organized the complete set of raw codes. Some authors did use the ICF core sets as reference standards typically stating what percentage of the items appeared on the core set. The complete set of indicators that we proposed which quantify how codes relate to ICF with specific definitions was used in 1 study by our group (33) and picked up by one other research group (61).

**Table 4:** Recent use of ICF linking in content validation.

**References**	**Tool/construct**	**Stage**	**Linking**	**Synthesis/analysis**	**Other methods/notes**
Lu et al. (33)	35 PROM identified by SR for total shoulder arthroplasty	EMCE	1 + LC 2005 2 + OM	RCM, CD, PL+, DSI	Definition of constructs e.g., QoL/health status
Roe et al. (42)	13 candidate PROMS for a shoulder core domain set	EMCE	2 + LC, LRR 2019 2 + OM	RCM, RCC, PL	Used perspective and response option rules
Osborne et al. (43)	Behavioral Assessment Screening Tool (BAST), a measure of behavioral disruptions after traumatic brain injury	IDIR	2 + LC LRR 2016 1OM	RCM, CD, RCC, PL	Use of conceptual model of construct
Wikström et al. (44)	Abilitator: work ability PROM	IDIR	2 + LC 2016 1OM	RCD, RCS, PL	7 stage mixed methods with iterative development and consultations described
Elvrum et al. (45)	Bimanual Fine Motor Function (BFMF) hand function	EMCE	2 + LC 2005 2OM	CD, RCC	Qualifiers capacity and performance were considered in the content
Carter et al. (46)	Leeds Foot Impact Scale in people with psoriatic arthritis	EMCE	2 + LC, LRR 20xx 1OM	CD, PLQ	Listed concepts not linkable in ICF
Ballert et al. (24)	41 participation measures that addressed at least three disability chapters of the ICF	EMCE	+LC 2019, 2 + OM, SS	CD, RCC, RO	Reported perspective
de Moraes et al. (47)	The Brachial Assessment Tool (BrAT) and the Impact of Brachial Plexus Injury Questionnaire (IBPIQ)	EMCE	2 + LC, LRR 2016 2 + OM, SS	RCM, RCC, CD	Did not report perspective and response options
Manchaiah et a. (48)	14 hearing loss PROMS	EMCE	2 + LC, LRR 2005, LM 2 + OM, SSS	RCM, PLC, PL	Personal factors coded with a different system; not codable reported
Darzins et al. (49)	Personal Care Participation Assessment and Resource Tool (PC-PART) and FIM (functional independence measure)	EMCE	2 + LC 2005 2 + OM	CD, RCM, RCC	Codes to 2nd level, ICF; FIM codes were extracted from published linking results; narrative comparative synthesis, informed by scenarios and discussion
Lassfolk et al. (50)	Spinal Function Sort and Functional Capacity Evaluation	EMCE	2 + LC, LRR 2016 2 + OM	CD, RCC, RCS, PL	PROM compared to performance tests
Oner et al. (51)	PROM and clinician-based outcome measures for spinal trauma	EMCE	2 + LC 2005 2 + OM, SS	RCM	Measures included if cited in at least 5 articles
Gutierrez et al. (52)	Military Concussion Readiness Inventory for Dizziness and Balance	IDIR	2 + LC 1OM	CD, RCM	Used formal consensus and other processes to inform development
Osborne et al. (43)	Pediatric Evaluation of Disability Inventory-Computer Adaptive Test (PEDI-CAT	EMCE	2 + LC, LRR 2014, LM 1OM	CD, RCC	Reported not linkable constructs; focused on links to chapters
Schiariti et al. (53)	42 PROM aligning with the ICF Core Sets for children and youth with cerebral palsy	EMCE	2005 2 + OM, SS	RCM, RCS	4 stage process to go from 80 + measures to 25
Burgess et al. (54)	8 upper limb activity measures for 5- to 18-year-old children with bilateral cerebral palsy	EMCE	1LC, 2016 2 + OM, SS	CD, RCC	Where publishing linking was found it was used; where no published data done by team, COSMIN used is synthesis; analysis of not codable items
Hammond et al. (55)	British English DASH	EMCE	Unclear linking process	RCS	Cross-cultural validation, Rasch
Janssen et al. (56)	32 PROMS for gout	EMCE	2 + LC 2016 2 + 2 + OM, SS	RCM, CD, RCC (in appended files)	did not link health concepts to the “other specified” or “unspecified” ICF categories; high content validity was assigned when ≥75% of the health concepts of the PROM were included on the ICF core set; used COSMIN criteria for content validity; did review of psychometric properties
Alam et al. (57)	Development of a PROM for abdominal surgery	IDIR	2 + LC 2005 1OM	RCM	Conceptual framework then Qualitative interview content linked to create outcome measure framework for item bank for CAT and standard PROM
Fresk et al. (58)	Test Instrument for Profile of Physical Ability	EMCE	2 + LC 2016 1OM	CD, RCM, RCS, RO	
Nund et al. (59)	27 Swallowing Outcome Measures for Head and Neck Cancer	EMCE	2 + LC, LRR 2005 2 + OM, SS	RCM, CD, RCC	
Lassfolk et al. (50)	26 migraine, tension-type headache, and cluster headache	EMCE	? raters 2 + OM, SS	CD, RCM, RCC, PL	Coded to 2^nd^ level not the most specific
Papelard et al. (60)	Activities and participation in patients with systemic sclerosis	IDIR	1LC 1OM	CD, RCM, RCC	Core set developed then questionnaires items created
Wong et al. (61)	The quality of life in neurological disorders (Neuro-QoL)	EMCE	1OM 2 + MC LRR 2002/2005	CD, RCC, DSI	Used the ICF linking indicators developed by MacDermid; attributed development to student author who used

*Stage: IDIR, Instrument Development Item Refinement; EMCE, Established Measure Content Evaluation*.

*Linking Process: Single linking coder, 1LC; 1 primary linking coder with calibration or checking process, 1 + LC; 2 or more linking coders, 2 + MC; Linking reliability reported, LRR; Year cited for linking rules, 1 PROM assessed, 1OM; 2 + PROM compared, 2 + OM; SS, systematic search used to identify measures*.

*Analysis: ICF linking Raw Code Map/Table, RCM; Concepts distribution (impairment, activity, participation, personal, and environmental factors), CD; ICF-linking Raw code comparison to Chapters, RCC; ICF-linking Raw code comparison to Core Set, RCS; Percentage of items linked to ICF/Core Set, PL; Defined Summary Indictors of linkage (MacDermid system), DSI*.

*Other: CI, Cognitive interviewing; CAT, computer adaptive test*.

## Discussion

This integrated narrative review illustrated the complexity of content validity, provides an operational definition, illustrates how ICF linking has been used to support description/mapping, and how cognitive interviewing complements ICF-linking. This review indicated the need for more consistent use of recent ICF rules, clear definitions of cognitive interview findings and better summary statistics to characterize findings of content validation. We provided definitions/classification to summarize sources of cognitive dissonance/interpretation errors derived from cognitive interviewing and simple statistics to summarize the results of ICF-linking to improve the consistency and interpretability of these methods in future content validity studies.

Ideally, content validity is integrated throughout development of a PROM and capitalizes on the knowledge and life experience of potential respondents, clinicians, and measurement experts. All will provide useful insights into how items and the entire PROM can be improved. In the past there was an overreliance on clinician experts in the PROM development process. The importance and methods for patient engagement improved as clinimetric methods evolved. Usually, the instrument developer/team defines a core construct needed in a PROM, and through patient engagement determines the item pool that represents that core construct, which is refined iteratively. ICF linking and qualitative methods should be considered as essential when developing PROM that address functioning, disability, and health. Our scoping review indicates that in most cases ICF linking has been used to evaluate PROMS already in use. This likely reflects the developing standards in content validity methods which were enhanced after many PROMS were already developed. This explains the need for retrospective content validation. The challenge in retrospective content validation is that “the horse is out of the barn” and changing an existing PROM can result in improvements, but also has downsides from version confusion and compromised data comparability. Less often ICF linking has been used to develop the conceptual framework for a construct that will be measured in a new PROM. This is ideal since it builds a strong foundation for the PROM. The emerging use of ICF linking during development indicates progress in awareness and implementation of formal methods for conducting, and reporting, content validity during PROM development. In our experience not all journals are interested in publishing content validation work done prior to establishing the final version of a PROM since they see this as preliminary work. However, given the importance of content validity it is important that these processes be documented. Further content validation is substantial piece of research and deserves a fulsome peer review and scientific discourse before PROMs are finalized.

Based on this narrative review we defined content validity as “*the extent to which a measure provides a comprehensive and true assessment of the key relevant elements of a specified construct or attribute across a defined range, clearly and equitably for a stated target audience and context*.” The definition is intended to be both conceptual and operational. It contains elements that can be assessed by different methodologies, including ICF linking. Concept mapping (62), qualitative description (63–66), content validity indices (6, 10, 12, 13, 36, 67–70), relevance surveys, focus groups, expert panels as examples of methods that might be used as part of the content validation process. These methods were not explored in this paper. The array of methods that inform content validity indicate that full rigorous evaluation of content validity should be possible with existing methods.

Validity is focused on whether a measure provides a true score. Although there are different types of validity, in the case of content validity “true” means that the items reflect the construct or attribute being measured “*Comprehensive, balanced*” and “*the key relevant elements of a specified construct or attribute*” focus on whether items provide a balanced assessment of the most relevant, important, or salient aspects of the attribute/construct being measured. Balance also refers to the emphasis placed on different aspects of the phenomenon, since the weighting of items should be directly proportional to how much those components contribute to the target construct. This is reflected in how many items are allocated to specific aspect of the construct being measured, and how that is reflected in score. For example, different PROM assess upper pain and disability differently based on how they weight pain and function. The Patient-Rated Wrist Evaluation weights 5 pain items (ICF code b278014) and 10 disability items at 50% and attributes this weighting to consultations with experts and the defined construct of this PROM (71, 72). The subscales are considered separately as subscales to separate these constructs (although often combined in a total score). The Disability, Arm, Shoulder Hand (73) PROM has 2/30 questions relate to pain and 3/30 that relate to other symptoms; these are summed and all items weighted equally in the total score. In these 2 examples the importance placed on pain is different and during content validity it might be considered if this is proportional to how important pain is to potential respondents with upper extremity conditions. ICF linking can be used to describe content of items, but also to summarize how content is weighted by assessing how often a specific code appears in relation to the total number of items. These aspects of content validation will support future structural validity of the PROM. If a subdomain is important, it may require multiple items. For example, since pain is a primary reason for seeking healthcare, it is often important to explore different dimensions of pain (ICF code b2780). These nuances would require multiple items that address different contexts in the stem (e.g., pain while doing an activity), perspectives, or dimensions e.g., frequency vs. intensity. With the updated ICF linking rules some of these nuances could now be reflected as perspective or response differences. There is a tension in “right-sizing” PROM since being comprehensive and minimizing respondent burden are conflicting goals. ICF linking can be used to help identify areas of overlap in content where efficiencies might be achieved is item reduction is needed.

Validity is tool, context, purpose, and population specific. The extent to which PROM items measure “*clearly and equitably for a stated target audience and context*” emphasizes the importance of considering personal factors like literacy, culture, language, gender, and socioeconomics of potential respondents on any PROM when developing or evaluating items. These differences can be explored in a variety of ways. Since ICF is a universal language embedded within a social view of health and functioning, it can be used as a start point to consider how items that are evaluating functioning might differ across contexts. Once an item is linked, the next question can be–would this aspect of functioning be similar for different populations, genders, or age groups? For example, the item “driving a car” is an item on the commonly used Neck Disability Index, but is also commonly left missing (74). People who cannot afford a car, who lived in countries where women are not allowed to drive cars, or who had their driving license taken away due to medical or age-related issues cannot answer this question. The NDI like many PROM was developed in North America where driving a car might be frequently mentioned as a problem for patients with neck pain. If the developers and used ICF in their thinking and item development, they might have taken a broader view. In ICF, d475 Driving falls under mobility (Chapter d4), and is defined as “*Being in control of and moving a vehicle or the animal that draws it, traveling under one's own direction or having at one's disposal any form of transportation appropriate for age, such as a car, bicycle, boat or animal powered vehicle.”* An ICF lens would have considered driving is often accomplished other ways outside of North America, and that the need to be the driver to achieve mobility across distances is far less important in some societies. Therefore, the functional intent of this item and the aggravation to neck conditions might be fulfilled by e d470 Using transportation- “Using *transportation to move around as a passenger, such as being driven in a car, bus, rickshaw, jitney, pram or stroller, wheelchair, animal-powered vehicle, private or public taxi, train, tram, subway, boat or aircraft and using humans for transportation.”* This illustrates how an ICF lens and ICF linking can prevent content validity problems that manifest later as missing items or flaws that show up during cross-cultural validation or psychometric studies. In our example, failure to take a broader functional view made the wording of an item on “driving” unnecessarily discriminatory.

An important addition to the 2019 linking rules that enhances the description of PROM focuses on item perspective and the response options. The added clarifications about how to link the perspective (appraisal, needs, or dependency) and response options (intensity, frequency, duration, confirmation or agreement, qualitative attributes) provides much better description of the nature and range of the assessments achieved by the items on a PROM. This aligns with the aspect of the content validity addressed in the definition by “across a defined range.” Clarity on the range where measures are accurate, whether it is a PROM or a biophysical tool, is important to avoid floor or ceiling effects. Floor/ceiling effects, interval level scaling (Rasch analysis) and factor analyses which might fall under structural validity provide more detailed assessment of the range and scoring metrics of a PROM. However, these assessments typically take place after PROMs are developed so it is important that content validity be thoughtfully designed and evaluated to support structural validity. The consequences of inadequate attention to content validity during development of a PROM is non-response, poor performance of the item in structural validation (factor analysis, Rasch) or other psychometric analyses.

Prior to the recent update which described how to assess perspective as part of ICF linking we had developed another process for describing perspective. We started by deciding whether items required rationale decisions e.g., how often do you do something, are you capable of performing, what is your pain intensity; or an emotional response e.g., satisfied with ability, fear of an event or outcome. Rationale decisions depend on calibrating past experiences, whereas emotive decisions reflect a current feeling that occurs in the moment of calibration. The perspectives identified in the refined ICF Linking Rules are the descriptive perspective (e.g., refers to the ability or the extent of a problem or difficulty a person experiences in performing a certain activity or task), appraisal (e.g., refers to the extent to which personal expectations and hopes have been achieved), and the perspective of needs or dependency (e.g., refers to how much assistive devices are needed to perform certain activities or tasks). Descriptive and appraisal definitions have overlap with the rationale and emotional definitions we used with ICF linking to describe health outcomes (75) and reflect some shared thinking about how to describe perspective in these independently developed perspective classifications. ICF linking of perspective is important since this can have a large impact on what construct is being measured. People with the same level of ability can have very different levels of satisfaction with their ability, and people with different levels of ability can achieve the same level of functioning using assistive devices. Understanding these nuances is important in understanding health outcomes, and particularly important in rehabilitation where both remediation and adaptation are important parts of the treatment process. In this study our scoping review confirmed that few authors have considered perspective as an important issue in item validation. The recent updates to the linking rules are important improvements that may facilitate greater attention to perspective and response options in future research.

The development of core sets has been invaluable for content validation and our review of methods in published studies indicated that use of the core sets as reference standards is emerging. The process of achieving international consensus on Core Sets makes them ideal reference standards. We found that authors using ICF-linking in content validation often created maps comparing the items on a PROM to the core set, in a large table. This is an important 1st step for looking at the congruence between items and the core set. Some use percentages to reflect this congruence. However, the ways these percentages were calculated was not consistent, which limits comparability across studies. The summary indicators we proposed which describe how to calculate indices of the linkage between PROM items and Core Sets provide consistent indicators that could be compared across studies or used to compare instruments within a study. For example, when we compared 2 similar elbow pain and disability PROM the summary indicators illustrated the high level of concordance in content between the two PROM (31). Conversely, when we used the indicators to compare different PROM used in total shoulder arthroplasty we found the summary indicators reflected very different profiles across PROM (33). We found that combining different approaches enhances the value of ICF-linking. For example in our shoulder arthroplasty outcomes study we explored how authors conceptualized measures as function or quality of life, which revealed a lack of clarity in conceptual frameworks and definitions (33). Our review of methods used by others indicated that it is common to augment ICF linking with other methods of content description such as conceptual definitions or codes for personal experience factors.

The strengths of ICF linking are the consistency and benefit of using a common language which provides a system to move from items to content codes. ICF linking provides detailed coding for content description. However, there are also gaps in what ICF linking provides in terms of content description, especially since not all PROM focus only on what ICF was designed to cover with respect to functioning, disability, and health. Some concepts are not definable by ICF (personal factors, emotions, abstract constructs, life experiences). For example, concepts like safety, fear of movement, happiness, optimism, negative thinking, prior life trauma and other constructs may not be ideally suited to ICF linking if they are not easily framed as an aspect of functioning. The most recent linking rule update provided clarity on how to code some non-specific or not definable item content assigning health in general, physical health or mental (emotional) health as not definable-general health, not definable-physical health, not definable-mental health (nd-gh, nd-ph, or nd-mh), respectively. Global content on disability in general (nd-dis), functioning (nd-func), or a child's development (nd-devdo) can be coded in a general sense as falling in these domains, even though not specifically definable (coded). While this allows for a code to be assigned, generic codes are not very helpful in content validation, especially when comparing different PROMs since the detail of the construct is lost. However, ICF does provide wide coverage of content and no single classification system could be expected to cover every potential thing that humans would want to measure in health research. The most recent linking rule update enhanced how ICF linking characterizes PROMs while maintaining the structural integrity and focus of the classification system. Although ICF recognizes that personal factors are important, these are not coded/classified. Others have add classification frameworks to address life experience (76) within PROM validation. However, no agreed upon classification for personal factors exists for content validation.

We propose that cognitive interviewing is the ideal complement to ICF linking in terms of providing a more comprehensive assessment of content validity. That is because whereas ICF linking focuses on content, cognitive interviewing focuses on how patients interpret and calibrate responses to that content. Together they provide a powerful assessment of what is being assessed by an outcome measure. Cognitive interviews were usually performed in person prior to the pandemic since it is useful to observe how the participants behave as they complete PROM items or as they “talk aloud” through their thinking. Since the pandemic many adaptations to research have been necessary, and video/web technologies can be used for cognitive interviews or other qualitative methods. As in other qualitative methods, probing in ways that encourage people to reveal their cognitive processes is a skill that is essential to optimize the quality of the information acquired. The processes involved in cognitive interviewing can produce a large volume of information and communicating the detail while also summarizing it to reveal key themes can be challenging. For this reason, we developed the Sources of *Cognitive Dissonance Classification System* comprised of definitions and a classification framework that were shared in this paper. Although ideally ICF linking and cognitive interviewing are used together, we did not find many papers where this occurred. This may be because researchers are not using both techniques together, and because they are reporting these findings separately in different manuscripts. We specifically focused on ICF linking in our search strategy and may have missed isolated cognitive interview studies. Since cognitive interviews and ICF linking are substantive pieces of work they may be published separately by some authors to ensure adequate attention to the rich information acquired. However, the integration of the findings across these two methods may be lost in separate publications. In such cases, authors need to ensure that the integration of these different studies into decisions about the final construction of a PROM or decisions about content validity of existing PROM is documented in manuals or other subsequent publications.

Content validation like many other aspects of instrument validation, often requires that multiple methods and studies be interpreted together when making decisions about PROM development or improvement. For example, we combined perspective classification portrayed in radar plots, ICF-linking table of raw codes, ICF concept description in a radar plot and ICF linkage indicators and their concordance with presenteeism core sets (32) to investigate the content of presenteeism (work disability) PROM. We found that most items mapped to a few ICF related work codes since work disability/presenteeism PROM focus on one specific type of participation. However, we were able to distinguish differences in PROMs by examining their perspectives, structures, and response options. Although this work was a preliminary step, gaps remain in our understanding of the content validity of presenteeism scales since conceptual frameworks that clarify the scope and components of the construct, content validity indices and qualitative studies are still lacking. This emphasizes that content validation is a process, not an event.

Overall, no single method or study is likely to give a full assessment of content validity. ICF linking is an important method in content validation which has many strengths, especially for health conditions where core sets have been developed and the focus of the PROM is on symptoms and functioning. It provides a rich content description language which can be used to describe item content and map PROMs to core sets and conceptual frameworks; and allows comparisons to be quantified within or across PROMs/studies. The major gap which remains after linking is how do patients engage with that content? That is best addressed though qualitative methods, preferably cognitive interviews, which are designed to explore how PROM items are understood and calibrated. While content validity has been under addressed in the literature, a common theme across existing studies is that humans, their contexts, and experiences are highly variable, and this is important to consider when designing or improving existing PROM. Methods for summarizing content validity findings in ICF linking and cognitive interviewing, as proposed in this paper, are helpful for analysis and scientific discourse about PROM content validity.

Although this paper provides insights into current content validation methods it is not a comprehensive of all methods that can be used. For example, quantitative methods like content validity indices (6, 10, 68, 70, 77, 78) (a survey method), concept/content mapping (62), mapping to consensus core sets (79), theoretical models other than ICF, clarification of conceptual models/construct definitions (80), or qualitative methods other than cognitive interviewing (69) were not explored in this paper. Given the array of appropriate methods that could be used in content validation, a prescriptive approach to content validation might lead to narrow thinking. Rather, a thoughtful and rigorous analysis of content validity using multiple methods is needed. Since our focus was ICF linking for content validation, we did not explore the many other uses of ICF linking including other types of work that have secondary impacts on what health constructs should be measured. Some of these related types of research include studies include studies that: code the disability experience (76), describe how the literature addresses disability (81), describe symptoms experienced by people living with different health conditions (63), or report the PROM used in clinical research within an ICF framework (33). Although we reviewed recent content validity papers to assess what methods are being currently used, we did not capture older papers, some of which were landmark papers that led thinking in this field. That is because wanted a snapshot of current content validation methods. We may have missed papers since some authors may have used terms that were not included in our search terms. Despite these limitations, our conclusion based on the retrieved sample of 24 recent studies is clear–there is wide variation in how ICF linking is used and the full spectrum of ICF linking rules and summary indicators are rarely reported by authors. While progress has been achieved on content validity methods, there is a need for full use of the updated linking, rules, better use of summary measures of content validation finings (as proposed in this paper), clear integration of qualitative and quantitative findings and more extensive reporting and public discourse on content validity during development of new PROM or modification of existing PROM.

## Author Contributions

The author confirms being the sole contributor of this work and has approved it for publication.

## Funding

JM was supported by a Canada Research Chair in Musculoskeletal Health Outcomes and Knowledge Translation and the Dr James Roth Chair in Musculoskeletal Measurement and Knowledge Translation. Her work was supported by a foundation grant from the Canadian Institutes of Health Research (#167284).

## Conflict of Interest

The author declares that the research was conducted in the absence of any commercial or financial relationships that could be construed as a potential conflict of interest.

## Publisher's Note

All claims expressed in this article are solely those of the authors and do not necessarily represent those of their affiliated organizations, or those of the publisher, the editors and the reviewers. Any product that may be evaluated in this article, or claim that may be made by its manufacturer, is not guaranteed or endorsed by the publisher.

## References

[1] Haynes SN Richard DCS Kubany ES. Content validity in psychological assessment: a functional approach to concepts and methods introduction to content validity. Psychol Assoc Sept. (1995) 7:238–47. 10.1037//1040-3590.7.3.238

[2] Beck CT Gable RK. Ensuring content validity: an illustration of the process. J Nurs Meas. (2001) 9:201–15. 10.1891/1061-3749.9.2.201 11696942

[3] Waltz CF Strickland OL Lenz ER. Measurement in Nursing and Health Research. New York, NY: Springer Publishing (2005).

[4] Wynd CA Schmidt B Schaefer MA. Two quantitative approaches for estimating content validity. West J Nurs Res. (2003) 25:508–18. 10.1177/0193945903252998 12955968

[5] Magasi S Ryan G Revicki D Lenderking W Hays RD Brod M. Content validity of patient-reported outcome measures: perspectives from a PROMIS meeting. Qual Life Res. (2012) 21:739–46. 10.1007/s11136-011-9990-8 21866374

[6] Polit DF Beck CT. The content validity index: are you sure you know what's being reported? Critique and recommendations. Res Nurs Health. (2006) 29:489–97. 10.1002/nur.20147 16977646

[7] Mokkink LB Terwee CB Patrick DL Alonso J Stratford PW Knol DL. The COSMIN study reached international consensus on taxonomy, terminology, and definitions of measurement properties for health-related patient-reported outcomes. J Clin Epidemiol. (2010) 63:737–45. 10.1016/j.jclinepi.2010.02.006 20494804

[8] Lennon RT. Assumptions underlying the use of content validity. Educ Psychol Meas. (1956) 16:294–304. 10.1177/001316445601600303

[9] Kanters DM Griffith LE Hogan DB Richardson J Patterson C Raina P. Assessing the measurement properties of a frailty index across the age spectrum in the Canadian longitudinal study on aging. J Epidemiol Community Health. (2017) 71:794–9. 10.1136/jech-2016-208853 28679540

[10] Patrick DL Burke LB Gwaltney CJ Leidy NK Martin ML Molsen E. Content validity - Establishing and reporting the evidence in newly developed patient-reported outcomes (PRO) instruments for medical product evaluation: ISPOR PRO good research practices task force report: Part 2 - Assessing respondent understanding. Value Health. (2011) 14:978–88. 10.1016/j.jval.2011.06.013 22152166

[11] Terwee CB Prinsen CAC Chiarotto A Westerman MJ Patrick DL Alonso J. COSMIN methodology for evaluating the content validity of patient-reported outcome measures: a Delphi study. Qual Life Res. (2018) 27:1159–70. 10.1007/s11136-018-1829-0 29550964 PMC5891557

[12] Bobos P MacDermid JC Boutsikari EC Lalone EA Ferreira L Grewal R. Evaluation of the content validity index of the Australian/Canadian osteoarthritis hand index, the patient-rated wrist/hand evaluation and the thumb disability exam in people with hand arthritis. Health Qual Life Outcomes. (2020) 18:1–9. 10.1186/s12955-020-01556-0 32907589 PMC7488300

[13] Yaghmale F. Content validity and its estimation. J Med Educ. (2003) 3:25–7.

[14] Wright JG Feinsten AR. A comparative contrast of clinimetric and psychometric methods for constructing indexes and rating scales. J Clin Epidemiol. (1992) 45:1201–18. 10.1016/0895-4356(92)90161-F 1432001

[15] Bellamy N Campbell J Haraoui B Buchbinder R Hobby K Roth JH. Dimensionality and clinical importance of pain and disability in hand osteoarthritis: development of the Australian/Canadian (AUSCAN) osteoarthritis hand index. Osteoarthr Cartil. (2002) 10:855–62. 10.1053/joca.2002.0837 12435330

[16] Rodrigues IB Adachi JD Beattie KA MacDermid JC. Development and validation of a new tool to measure the facilitators, barriers and preferences to exercise in people with osteoporosis. BMC Musculoskelet Disord. (2017) 18:540. 10.1186/s12891-017-1914-5 29258503 PMC5738121

[17] Willis GB. Cognitive Interviewing a Tool for Improving Questionnaire Design. Thousand Oaks, CA: Sage Publications (2004). 10.1525/jer.2006.1.1.9

[18] Packham T MacDermid JC Henry J Bain JR. The hamilton inventory for complex regional pain syndrome: a cognitive debriefing study of the clinician-based component. J Hand Ther. (2012) 25:97–112. 10.1016/j.jht.2011.09.007 22265445

[19] Fayed N Cieza A Bickenbach JE. Linking health and health-related information to the ICF: a systematic review of the literature from 2001 to 2008. Disabil Rehabil. (2011) 33:1941–51. 10.3109/09638288.2011.553704 21303198

[20] Weigl M Cieza A Harder M Geyh S Amann E Kostanjsek N. Linking osteoarthritis-specific health-status measures to the international classification of functioning, disability, and health (ICF). Osteoarthr Cartil. (2003) 11:519–23. 10.1016/S1063-4584(03)00086-4 12814615

[21] Cieza A Geyh S Chatterji S Kostanjsek N Üstün B Stucki G. ICF linking rules: an update based on lessons learned. J Rehabil Med. (2005) 37:212–8. 10.1080/16501970510040263 16024476

[22] Cieza A Fayed N Bickenbach J Prodinger B. Refinements of the ICF linking rules to strengthen their potential for establishing comparability of health information. Disabil Rehabil. (2019) 41:574–83. 10.3109/09638288.2016.1145258 26984720

[23] Cieza A Brockow T Ewert T Amman E Kollerits B Chatterji S. Linking health-status measurements to the international classification of functioning, disability and health. J Rehabil Med. (2002) 34:205–10. 10.1080/165019702760279189 12392234

[24] Ballert CS Hopfe M Kus S Mader L Prodinger B. Using the refined ICF linking rules to compare the content of existing instruments and assessments: a systematic review and exemplary analysis of instruments measuring participation. Disabil Rehabil. (2019) 41:584–600. 10.1080/09638288.2016.1198433 27414962

[25] Coenen M Kus S Rudolf KD Müller G Berno S Dereskewitz C. Do patient-reported outcome measures capture functioning aspects and environmental factors important to individuals with injuries or disorders of the hand? J Hand Ther. (2013) 26:332–42. 10.1016/j.jht.2013.06.002 23911076

[26] Stucki G Ewert T Cieza A. Value and application of the ICF in rehabilitation medicine. Disabil Rehabil. (2002) 24:932–8. 10.1080/09638280110070221 12523361

[27] Stucki G Cieza A Melvin J. The international classification of functioning, disability and health: a unifying model for the conceptual description of the rehabilitation strategy. J Rehabil Med. (2007) 39:279–85. 10.2340/16501977-0041 17468799

[28] WHO. Towards a Common Language for Functioning, Disability Health ICF. WHO/EIP/GPE/CAS/01.3. Geneva: WHO (2002).

[29] Gargon E Williamson PR Young B. Improving core outcome set development: qualitative interviews with developers provided pointers to inform guidance. J Clin Epidemiol. (2017) 86:140–52. 10.1016/j.jclinepi.2017.04.024 28495644 PMC5513443

[30] Yen TH Liou TH Chang KH Wu NN Chou LC Chen HC. Systematic review of ICF core set from 2001 to 2012. Disabil Rehabil. (2013) 36:1–8. 10.3109/09638288.2013.782359 23651126

[31] Vincent JI MacDermid JC King GJW Grewal R. Linking of the patient rated elbow evaluation (PREE) and the American shoulder and elbow surgeons - elbow questionnaire (pASES-e) to the international classification of functioning disability and health (ICF) and hand core sets. J Hand Ther. (2015) 28:61–8. 10.1016/j.jht.2014.10.002 25727010

[32] Arumugam V MacDermid JC Grewal R. Content analysis of work limitation, stanford presenteeism, and work instability questionnaires using international classification of functioning, disability, and health and item perspective framework. Rehabil Res Pract. (2013) 2013:614825. 10.1155/2013/614825 24459587 PMC3888761

[33] Lu Z MacDermid JC Rosenbaum P. A narrative review and content analysis of functional and quality of life measures used to evaluate the outcome after TSA: an ICF linking application. BMC Musculoskelet Disord. (2020) 21:1–11. 10.1186/s12891-020-03238-w 32284065 PMC7155280

[34] Wright J Moghaddam N Dawson DL. Cognitive interviewing in patient-reported outcome measures: a systematic review of methodological processes. Qual Psychol. (2019) 8:2–29. 10.1037/qup0000145

[35] Willis GB. Cognitive interviewing revisited: a useful technique, in theory? Methods Test Eval Surv Quest. (2004) 23–42. 10.1002/0471654728.ch2

[36] Haynes SN Richard DCS Kubany ES. Content validity in psychological assessment: a functional approach to concepts and methods. Psychol Assess. (1995) 7:238–47. 10.1037/1040-3590.7.3.238

[37] Blair J Conrad FG. Sample size for cognitive interview pretesting. Public Opin Q. (2011) 75:636–58. 10.1093/poq/nfr035

[38] Chiarotto A Ostelo RW Boers M Terwee CB. A systematic review highlights the need to investigate the content validity of patient-reported outcome measures for physical functioning in patients with low back pain. J Clin Epidemiol. (2018) 95:73–93. 10.1016/j.jclinepi.2017.11.005 29154811

[39] Shafer K Lohse B. How to Conduct a Cognitive Interview: Nutrition Education Example. Training Manual (2005). p. 1–36. Available online at: https://nifa.usda.gov/sites/default/files/resource/how-to-conducta-cognitive-interview.pdf

[40] Packham TL MacDermid JC Michlovitz SL Buckley N. Content validation of the patient-reported hamilton inventory for complex regional pain syndrome. Can J Occup Ther. (2018) 85:99–105. 10.1177/0008417417734562 29475370

[41] MacDermid JC. Cognitive Interviewing (CI) - to Identify Sources of Interpretation Dissonance in in Patient-Reported Outcome Measures (PROM). Available online at: https://www.lawsonresearch.ca/hulc/tools-or-products-research

[42] Røe Y Buchbinder R Grotle M Whittle S Ramiro S Huang H. What do the OMERACT shoulder core set candidate instruments measure? An analysis using the refined international classification of functioning, disability, and health linking rules. J Rheumatol. (2020) 47:1557–64. 10.3899/jrheum.190832 32062599

[43] Osborne CL Kauvar DS Juengst SB. Linking the behavioral assessment screening tool to the international classification of functioning, disability, and health as a novel indicator of content validity. Disabil Rehabil. (2020) 42:1775–82. 10.1080/09638288.2018.1539128 30656977

[44] Wikström M Anttila H Savinainen M Kouvonen A Joensuu M. Development and content validity of the abilitator: a self-report questionnaire on work ability and functioning aimed at the population in a weak labour market position. BMC Public Health. (2020) 20:327. 10.1186/s12889-020-8391-8 32171263 PMC7071596

[45] Elvrum A-KG Andersen GL Himmelmann K Beckung E Öhrvall AM Lydersen S Vik T. Bimanual fine motor function (BFMF) classification in children with cerebral palsy: aspects of construct and content validity. Phys Occup Ther Pediatr. (2016) 36:1–16. 10.3109/01942638.2014.975314 25374154

[46] Carter K Tannous C Walmsley S Rome K Turner DE. Linking the effect of psoriatic arthritis-related foot involvement to the leeds foot impact scale using the international classification for functioning, disability and health: a study to assess content validity. J Foot Ankle Res. (2020) 13:52. 10.1186/s13047-020-00420-0 32831126 PMC7445917

[47] de Moraes AA de Dantas DS de Chagas ACS de Melo PH de Oliveira DA. Linking assessment instruments for brachial plexus injury to the international classification of functioning, disability and health. J Hand Ther. (in press). 10.1016/J.JHT.2021.04.009 34247880

[48] Manchaiah V Granberg S Grover V Saunders GH Ann Hall D. Content validity and readability of patient-reported questionnaire instruments of hearing disability. Int J Audiol. (2019) 58:565–75. 10.1080/14992027.2019.1602738 31017493

[49] Darzins SW Imms C Di Stefano M. Measurement of activity limitations and participation restrictions: examination of ICF-linked content and scale properties of the FIM and PC-PART instruments. Disabil Rehabil. (2017) 39:1025–38. 10.3109/09638288.2016.1172670 27206817

[50] Lassfolk M Escorpizo R Korniloff K Reneman M. Linking the spinal function sort and functional capacity evaluation tests to the international classification of functioning, disability and health core set of vocational rehabilitation. J Occup Rehabil. (2021) 31:166–74. 10.1007/s10926-020-09905-y 32500472

[51] Oner FC Jacobs WCH Lehr AM Sadiqi S Post MW Aarabi B. Toward the development of a universal outcome instrument for spine trauma: a systematic review and content comparison of outcome measures used in spine trauma research using the ICF as reference. Spine. (2016) 41:358–67. 10.1097/BRS.0000000000001207 26555824

[52] Gutierrez ML Christy JB Whitney SL. Development of military concussion readiness inventory for dizziness and balance. Patient Relat Outcome Meas. (2019) 10:67–80. 10.2147/PROM.S171380 30881164 PMC6398398

[53] Schiariti V Tatla S Sauve K O'Donnell M. Toolbox of multiple-item measures aligning with the ICF core sets for children and youth with cerebral palsy. Eur J Paediatr Neurol. (2017) 21:252–63. 10.1016/j.ejpn.2016.10.007 27864012

[54] Burgess A Boyd RN Ziviani J Sakzewski L. A systematic review of upper limb activity measures for 5- to 18-year-old children with bilateral cerebral palsy. Aust Occup Ther J. (2019) 66:552–67. 10.1111/1440-1630.12600 31385319

[55] Hammond A Prior Y Tyson S. Linguistic validation, validity and reliability of the british english versions of the disabilities of the arm, shoulder and hand (DASH) questionnaire and QuickDASH in people with rheumatoid arthritis. BMC Musculoskelet Disord. (2018) 19:118. 10.1186/s12891-018-2032-8 29661183 PMC5902839

[56] Janssen CA Oude Voshaar MAH ten Klooster PM Jansen TLTA Vonkeman HE van de Laar MAFJ. A systematic literature review of patient-reported outcome measures used in gout: an evaluation of their content and measurement properties. Health Qual Life Outcomes. (2019) 17:63. 10.1186/s12955-019-1125-x 30975212 PMC6460780

[57] Alam R Montanez J Law S Lee L Pecorelli N Watanabe Y. Development of a conceptual framework of recovery after abdominal surgery. Surg Endosc. (2020) 34:2665–74. 10.1007/s00464-019-07044-x 31372888

[58] Fresk M Brodin N Grooten WJ Joseph C Kiessling A. Mapping a measure of physical ability for persons with long-term musculoskeletal pain to the ICF and ICF core sets. Eur J Public Health. (2019) 29:286–91. 10.1093/eurpub/cky135 30085005

[59] Nund RL Brown B Ward EC Maclean J Roe J Patterson JM. What are we really measuring? A content comparison of swallowing outcome measures for head and neck cancer based on the international classification of functioning, disability and health (ICF). Dysphagia. (2019) 34:575–91. 10.1007/s00455-019-10005-0 30945002

[60] Papelard A Daste C Alami S Sanchez K Roren A Segretin F. Construction of an ICF core set and ICF-based questionnaire assessing activities and participation in patients with systemic sclerosis. Rheumatology. (2019) 58:2260–72. 10.1093/rheumatology/kez209 31219594

[61] Wong AWK Lau SCL Cella D Lai JS Xie G Chen L. Linking of the quality of life in neurological disorders (Neuro-QoL) to the international classification of functioning, disability and health. Qual Life Res. (2017) 26:2435–48. 10.1007/s11136-017-1590-9 28477085

[62] Rosas SR Camphausen LC. The use of concept mapping for scale development and validation in evaluation. Eval Program Plann. (2007) 30:125–35. 10.1016/j.evalprogplan.2007.01.003 17689319

[63] MacDermid JC Walton DM Bobos P Lomotan M Carlesso L. A qualitative description of chronic neck pain has implications for outcome assessment and classification. Open Orthop J. (2016) 10:746–56. 10.2174/1874325001610010746 28217199 PMC5301418

[64] Sandelowski M. Focus on research methods: whatever happened to qualitative description? Res Nurs Health. (2000) 23:334–40. 10.1002/1098-240x(200008)23:4<334::aid-nur9>3.0.co;2-g 10940958

[65] Tong A Sainsbury P Craig J. Consolidated criteria for reporting qualitative research (COREQ): a 32-item checklist for interviews and focus groups. Int J Qual Heal Care. (2007) 19:349–57. 10.1093/intqhc/mzm042 17872937

[66] Thorne S. Cap 3. Interpretive Description. Walnut Creek, CA: Left Coast Press Inc. (2008).

[67] Ailliet L Knol DL Rubinstein SM De Vet HCW Van Tulder MW Terwee CB. Definition of the construct to be measured is a prerequisite for the assessment of validity. The neck disability index as an example. J Clin Epidemiol. (2013) 66:775–82. 10.1016/j.jclinepi.2013.02.005 23618795

[68] Yalow ES Popham WJ. Content validity at the crossroads. Educ Res. (1983) 12:10–21. 10.3102/0013189X012008010

[69] Zamanazedeh V Rassouli M Abbaszadeh A Majd HA Nikanfar A Ghahramanian A. Details of content validity and objectifying it in instrument development. Nurs Pract Today. (2014) 1:163–71.

[70] Lawshe CH. A quantitative approach to content validity. Pers Psychol. (1975) 28:563–75. 10.1111/j.1744-6570.1975.tb01393.x

[71] MacDermid JC. Development of a scale for patient rating of wrist pain and disability. J Hand Ther. (1996) 9:178–83. 10.1016/S0894-1130(96)80076-7 8784681

[72] MacDermid JC Turgeon T Richards RS Beadle M Roth JH. Patient rating of wrist pain and disability: a reliable and valid measurement tool. J Orthop Trauma. (1998) 12:577–86. 10.1097/00005131-199811000-00009 9840793

[73] Beaton DE Katz JN Fossel AH Wright JG Tarasuk V Bombardier C. Measuring the whole or the parts? Validity, reliability and responsiveness of the disabilities of the arm, shoulder and hand outcome measure in different regions of the upper extremity. J Hand Ther. (2001) 14:128–46. 10.1016/S0894-1130(01)80043-0 11382253

[74] MacDermid JC Walton DM Avery S Blanchard A Etruw E McAlpine C. Measurement properties of the neck disability index: a systematic review. J Orthop Sports Phys Ther. (2009) 39:400–17. 10.2519/jospt.2009.2930 19521015

[75] Rosa D Macdermid J Klubowicz D. A comparative performance analysis of the international classification of functioning, disability and health and the item-perspective classification framework for classifying the content of patient reported outcome measures. Health Qual Life Outcomes. (2021) 19:132. 10.1186/s12955-021-01774-0 33892735 PMC8066430

[76] Geyh S Schwegler U Peter C Muller R. Representing and organizing information to describe the lived experience of health from a personal. Disabil Rehabil. (2019) 41:1727–38. 10.1080/09638288.2018.1445302 29509044

[77] Aiken LR. Content validity and reliability of single items or questionnaires. Educ Psychol Meas. (1980) 40:955–9.

[78] Delgado-rico E Carretero-dios H Ruch W. Content validity evidences in test development. Int J Clin Health Psychol. (2012) 12:449–59. 10.4090/juee.2009.v3n2.052057

[79] Crudgington H Collingwood A Bray L Lyle S Martin R Gringras P. Mapping epilepsy-specific patient-reported outcome measures for children to a proposed core outcome set for childhood epilepsy. Epilepsy Behav. (2020) 112:107372. 10.1016/j.yebeh.2020.107372 32906016 PMC7689576

[80] Yong J MacDermid JC Packham T. Defining dexterity—Untangling the discourse in clinical practice. J Hand Ther. (2020) 33:517–9. 10.1016/j.jht.2019.11.001 31956020

[81] Thomson L Fayed N Sedarous F Ronen GM. Life quality and health in adolescents and emerging adults with epilepsy during the years of transition: a scoping review. Dev Med Child Neurol. (2014) 56:421–33. 10.1111/dmcn.12335 24237329

