# Supplementary material for: ICF Linking and Cognitive Interviewing Are Complementary Methods for Optimizing Content Validity of Outcome Measures: An Integrated Methods Review
Source: Front Rehabil Sci. 2021 Oct 14;2:702596. doi: 10.3389/fresc.2021.702596 (PMC9397968; doi:10.3389/fresc.2021.702596)
Supplement: Supplementary file 1 [file Data_Sheet_1.pdf]

Appendix 1 . ICF LINKAGE INDICATOR DEFINITIONS

Author: JC MacDermid

Raters can describe the content of an outcome measure using the instructions/training and established linking rules ([2–4]) and any further updates established by the ICF branch to select the ICF codes that best represent the content of items/measures. This content coding can then be summarized by the following indicators that compare the content items to the ICF or its subset Core Sets where these exist.

These measures summarize codable content. Only codes are counted; "not codable" codes are reported, but not included in the summary indicators below. It is useful to describe the number of codes and the distribution, e.g., by chapters or domains, in addition to the summary terms below. These are intended to be descriptive summary measures that reflect a perspective on measures and their relationship to ICF overall, and to core sets, but should be used in combination with other descriptive strategies to fully describe or compare measures.

1) Measure to ICF linkage:

This is the percentage of items from a measure that can be linked to ICF codes. This represents the extent to which content of a measure can be expressed in ICF codes.

Measure to ICF linkage $= \frac{\text{The number of items linked to at least 1 ICF code}}{\text{Total number of items on the measure}} \times 100\%$

2) Measure to (Brief or Comprehensive) Core Set Absolute Linkage

This is the percentage of items from a measure that could be linked to ICF codes that appear on a relevant Brief or Comprehensive Core Set.

$$= \frac{\text{Number of items linked to a code(s) appearing in the CoreSet}}{\text{Total number of items on the measure}} \times 100\%$$

3) Measure to (Brief or Comprehensive) Core Set Unique Linkage:

This is the percentage of items from a measure that could be linked to **unique** ICF codes and represents the extent to which the items of a measure represent different content from the core set. Once an item is coded to a core set item, additional items that code to that same code are not counted again.

$$= \frac{\text{Number of items that are linked to Unique codes in Core Set}}{\text{Total number of items on the scale}} \times 100$$

4) Core Set Representation

This is the percentage of core set codes that are covered when the measure's items are linked to ICF codes. This represents the extent to which the entire scope of content defined by the core set is represented on the measure.

$$= \frac{\text{Number of unique ICF codes from the measure that appear in the CoreSet}}{\text{Total number of codes in the (Brief or Comprehensive) CoreSet}} \times 100\%$$

5) Core Set Unique Disability Representation:

This is the percentage of unique core set disability codes that are covered when the measure's items are linked to ICF codes. For Patient-Reported Outcome (PROs) Measures that were designed to measure disability, it can be important to determine the extent to which they measure this aspect of content. This represents the extent to which the disability codes defined by the core set are represented on the measure. Once an item is coded to a core set disability code, additional items that code to that same code are not counted again.

$$= \frac{\text{Number of unique (d)codes from the measure that appear in the Core Set}}{\text{Total number of disability codes in the (Brief or Comprehensive) CoreSet}} \times 100\%$$

Example publications that have used these indicators[1, 5, 6] .

1. Arumugam V, Macdermid JC, Grewal R. Content analysis of work limitation, stanford presenteeism, and work instability questionnaires using international classification of functioning, disability, and health and item perspective framework. *Rehabil Res Pract*. 2013;2013:614825. Available at: http://www.pubmedcentral.nih.gov/articlerender.fcgi?artid=3888761&tool=pmcentrez&rendertype=abstract.

2. Cieza A, Brockow T, Ewert T, Amman E, Kollerits B, Chatterji S, Üstün TB, Stucki G. Linking health-status measurements to the International Classification of Functioning, Disability and Health. *J Rehabil Med*. 2002;34:205-210.

3. Cieza A, Fayed N, Bickenbach J, Prodinger B. Refinements of the ICF Linking Rules to strengthen their potential for establishing comparability of health information. *Disabil Rehabil*. 2019;41:574-583.

4. Cieza A, Geyh S, Chatterji S, Kostanjsek N, Üstün B, Stucki G. ICF linking rules: An update based on lessons learned. *J Rehabil Med*. 2005;37:212-218.

5. Lu Z, MacDermid JC, Rosenbaum P. A narrative review and content analysis of functional and quality of life measures used to evaluate the outcome after TSA: an ICF linking application. *BMC Musculoskelet Disord*. 2020;21:1-11.

6. Vincent JI, Macdermid JC, King GJW, Grewal R. Linking of the Patient Rated Elbow Evaluation (PREE) and the American Shoulder and Elbow Surgeons - Elbow questionnaire (pASES-e) to the International Classification of Functioning Disability and Health (ICF) and hand core sets. *J Hand*

Ther. 2015;28:61-68.
